# Improvement in quality of life and general functions in pediatric acid sphingomyelinase deficiency patients after receiving olipudase alfa: A single-center experience in Taiwan

**DOI:** 10.1016/j.ymgmr.2026.101312

**Published:** 2026-04-17

**Authors:** Yu-Wen Pan, Wen-Hao Yu, Meng-Che Tsai, Yen-Yin Chou

**Affiliations:** Department of Pediatrics, National Cheng Kung University Hospital, College of Medicine, No. 138, Shengli Rd., North Dist., Tainan City, Taiwan

**Keywords:** ASMD, ERT, Quality of life, Functional capability

## Abstract

**Background:**

The ultra-rare lysosomal storage disease, Acid sphingomyelinase deficiency (ASMD), is currently managed with olipudase alfa, an enzyme replacement therapy (ERT). Specifically targeting the non-neurological manifestations in chronic ASMD cases, olipudase alfa has demonstrated efficacy in reducing disease burdens through clinical trials and real-world studies. However, data on the impact of olipudase alfa on quality of life and its real-world influences are limited.

**Materials and methods:**

By May 2024, two pediatric patients-Patient 1 (P1) and Patient 2 (P2)-diagnosed with chronic neurovisceral ASMD A/B had undergone three years of olipudase alfa treatment in a single tertiary medical center in southern Taiwan. Throughout the treatment course, major outcomes including neurocognitive function and functional capability assessments were measured regularly. The primary caregivers completed the PedsQL™ version 4.0 questionnaire for the evaluation of the quality of life of the patients. Additionally, the progress of major symptoms was independently assessed using a customized questionnaire.

**Results:**

Following treatment with olipudase alfa, the six-minute walking distance improved from zero to 127.7 m in P1 and from zero to 340 m in P2. Functional capability, measured by the EDSS and pmRS scales, showed no decline in either patient. Quality of life, evaluated using the PedsQL 4.0 generic scales, which measures the frequency of discomfort caused by the disease, showed improvement for both patients. The total score decreased from 64 to 55 in P1 and from 58 to 39 in P2, with the greatest improvement observed in physical functioning. Among the major symptoms and signs, abdominal fullness and growth failure showed the most notable improvements. However, the caregiver noted increased emotional challenges in P1, particularly due to fear of needle injections following treatment. For P1, the full-scale IQ score fell from the 0.2nd percentile at baseline to below the 0.1st percentile after three years of enzyme replacement therapy (ERT). In P2, Bayley III scores for cognitive, language, and motor domains remained below the 1st percentile compared to age- and gender-matched controls, showing no obvious improvement or deterioration after treatment.

**Conclusion:**

ERT improves functional capacity and quality of life in two pediatric patients with chronic neurovisceral ASMD A/B. Further data from larger samples are required to confirm these findings.

## 1 Introduction

Acid sphingomyelinase deficiency (ASMD), traditionally known as Niemann-Pick disease type A, A/B and B, is a rare lysosomal storage disease (LSD). It is caused by bi-allelic disease-causing variants of *SMPD1* gene which encodes acid-sphingomyelinase, an enzyme responsible for the breakdown of sphingomyelin [1]. Insufficient or complete absence of enzyme activity results in the accumulation of sphingomyelin in multiple vital organs. The typical presentations encompass hepatosplenomegaly, interstitial lung disease, cytopenia, dyslipidemia, growth failure with or without neurological deficits. There is a broad spectrum of clinical severity, ranging from the most rapidly progressing neurodegenerative infantile neurovisceral ASMD (Niemann-Pick disease type A) to chronic neurovisceral ASMD (Niemann-Pick disease type A/B) and chronic visceral ASMD (Niemann-Pick disease type B) without neurologic involvement. The latter two are categorized as chronic ASMD [2]. Olipudase alfa, the first disease-modifying therapy for ASMD patients, functions as enzyme replacement therapy (ERT) [3]. Clinical trials involving both adults and children have demonstrated the efficacy of ERT in improving major ASMD outcomes, such as liver/spleen volume, interstitial lung disease score, biomarker levels, and lipid profiles [[4], [5], [6], [7]]. Based on the findings from clinical trials, olipudase alfa received FDA approval in 2022 for the treatment of non-CNS manifestations in ASMD patients across all age groups in Taiwan. While limited data is available, there have been observations of real-world impact, such as improvements in symptoms and quality of life for ASMD patients and families after ERT [8]. Therefore, the objective of this study is to conduct a more comprehensive evaluation of olipudase alfa's influence on general functional capabilities during daily life and quality of life in real-world settings.

## 2 Materials and methods

**Fig. 1:**
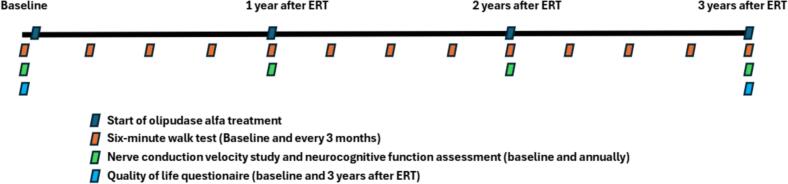
Timeline of scheduled assessments throughout the study period.

Two unrelated pediatric patients diagnosed with chronic neurovisceral ASMD A/B at National Cheng-Kung University Hospital (NCKUH) have received olipudase alfa through a compassionate use program since 2021. Treatment was initiated at 0.03 mg/kg and titrated to 3 mg/kg over 16 weeks according to the dose-escalation protocol, followed by maintenance at the full dose for three years without severe adverse events (AEs). By May 2024, both patients had received ERT for three years. To evaluate the real-world impact of ERT, we performed longitudinal assessments of neurocognitive function, peripheral neuropathy, and functional capacity at baseline (when only supportive care had been provided) and at predefined intervals following ERT initiation. Quality of life was assessed using a questionnaire-based approach, reflecting the overall impact of the disease and its treatment on daily functioning and well-being. This study was approved by the Ethics Committee of National Cheng-Kung University Hospital (approval number: A-ER-113-391). Informed consents were obtained from the legal guardians of the patients. The assessment tools are listed below, and the corresponding time points are shown in Fig. 1.

### 2.1 Neurocognitive function (CNS)

Neurocognitive function was assessed using age-appropriate instruments: the Wechsler Preschool and Primary Scale of Intelligence, 4th Edition (WPPSI-IV) or the Wechsler Intelligence Scale for Children, 5th Edition (WISC-V) for P1, and the Bayley Scales of Infant and Toddler Development, 3rd Edition (Bayley-III) for P2.The assessments were performed at baseline and at 1, 2, and 3 years after ERT initiation.

### 2.2 Peripheral neuropathies

Both patients underwent serial nerve conduction velocity (NCV) tests annually to assess the presence and severity of peripheral neuropathy at baseline and at 1, 2, and 3 years after ERT initiation.

### 2.3 Functional capability

#### 2.3.1 Six-minute walk test (6MWT)

The six-minute walk test (6MWT) was performed at baseline and every 3 months after ERT initiation. The patient was instructed to walk back and forth between two marked points along a designated hallway. The total walking distance within six minutes was recorded. Vital signs were monitored at baseline, the second minute, and upon completion of the test.

#### 2.3.2 Expanded disability status scale (EDSS)

The Expanded Disability Status Scale (EDSS) is a standardized tool used to quantify disability in multiple sclerosis (MS) and has also been applied to pediatric patients with other lysosomal storage diseases [9]. The scale ranges from 0 to 10, with half-point increments. Lower scores indicate less disability, while higher scores indicate greater disability.

#### 2.3.3 Pediatric modified Rankin scale (pmRS)

The Pediatric Modified Rankin Scale (pmRS), adapted from the original Modified Rankin Scale (mRS), is used to assess disability in children who have experienced neurological events, taking age-appropriate activities into account [10]. The scale ranges from 0 to 6, with one-point increments, indicating varying degrees of disability or independence. A higher score on this scale indicates more severe disability.

A single experienced pediatric neurologist evaluated the EDSS and pmRS at baseline and at 1, 2, and 3 years after ERT initiation. The evaluations were based on neurological examination findings and multiple domains of disability, including mobility, coordination, sensory function, bowel and bladder function, vision, cognition, and dependence in activities of daily living.

### 2.4 Quality of life

To evaluate the impact of ERT on the quality of life of children with chronic neurovisceral ASMD A/B, the primary caregiver was asked to recall the baseline condition prior to treatment and to complete the parent-proxy version of the PedsQL™ 4.0 questionnaire at baseline and after 3 years of ERT [11]. This questionnaire is widely used to assess the health-related quality of life (HRQOL) in children and adolescents, offering both self-report and proxy-report formats. It encompasses four domains- physical, emotional, social, and school functioning, comprising 8, 5, 5, and 5 items, respectively. Each item is individually scored to gauge the frequency of daily life difficulties described. Additionally, symptom changes, as described in the study conducted by Raebel et al. [8], were assessed using self-designed questionnaires. Higher scores on both questionnaires indicate a greater frequency of daily difficulties and a lower quality of life. The 23-item PedsQL 4.0 Generic Core Scales were initially developed for general use across various pediatric populations. It has been utilized in studies involving specific medical conditions, including LSDs such as Gaucher disease [12].

## 3 Results

The clinical and genotypic information were listed in Table 1.

**Table 1:** ASMD patient characteristics.

	Gender	Ethnicity	Age at symptoms onset	Age at diagnosis	Age at treatment start	*SMPD1* variant	Clinical manifestations
P1	Male	Taiwanese	<3y	3y2m	5y8m	c.1486 + 5G > C(pat) c.1498 T > C (Y500H)(mat)	Hepatosplenomegaly Mildly elevated liver function Growth failure Interstitial lung disease Developmental delay
P2	Male	Taiwanese	<1y	1y11m	2y6m	c.1498 T > C (Y500H)(pat) c.1486 + 5G > C(mat)	Hepatosplenomegaly Mildly elevated liver function Growth failure Interstitial lung disease Repeated bronchopneumonia episodes Bilateral cherry-red spots Developmental delay

mat: maternal; pat: paternal.

### 3.1 Neurological function

In P1, full-scale IQ scores, measured using the WPPSI-IV, were 56 at baseline (0.2nd percentile) and 50 at week 50 (below the 0.1st percentile). By week 132, the full-scale IQ score measured using the WISC-V had declined to 44 (below the 0.1st percentile). P2's Bayley-III cognitive, language, and motor scores remained below the 0.1st percentile both before and after ERT. Overall, neither patient showed noticeable improvement in neurocognitive function. Additionally, serial NCV studies conducted before and after treatment showed no significant improvement, as presented in the supplemental data (additional file 1).

### 3.2 Functional capabilities

**Fig. 2:**
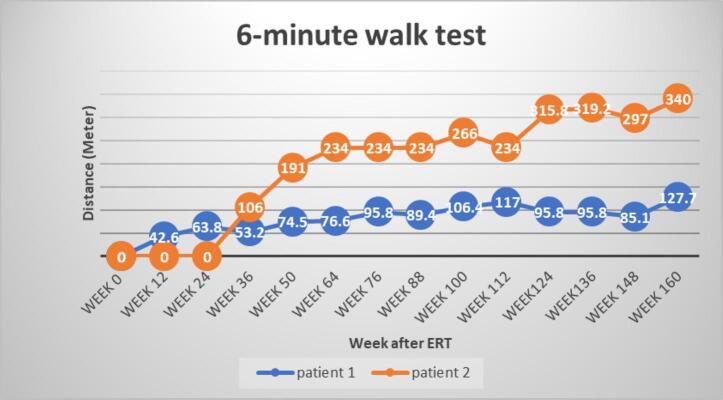
Both patients were unable to complete the 6-min walk test (6MWT) at baseline. P1 was physically weak due to a gastrointestinal illness that caused severe abdominal distension. P2 had developmental delays and was too young to perform the test during the first six months. Although he was able to walk with assistance in daily life, he was unable to cooperate with the assessments. As a result, his walking distance was recorded as zero at weeks 0 (baseline), 12, and 24. By week 36 after treatment initiation, as he became more familiar with the environment, he was willing to cooperate with the test. Following treatment, the 6-min walking distance increased for both patients. P1 improved to 74.5 m (week 50) during the first year but did not show further improvement in the subsequent two years. In contrast, P2 exhibited significant enhancements, progressing from 191 m (week 50) in the first year to 234 m (week 112) in the second year and 340 m in the third-year post-treatment (week 160). The walking distance data presented here extends to the most recent six-minute walk test (6MWT) that the patients underwent.

The 6-min walking distances showed significant improvements after ERT for both patients (Fig. 2). The most notable increase occurred during the first year, with P1 improving by 74.5 m and P2 by 191 m. For P2, the walking distance continued to improve in the second and third year, with increases of 75 m and 53 m, respectively. However, P1 did not show obvious improvements or deterioration in the subsequent two years.

**Fig. 3:**
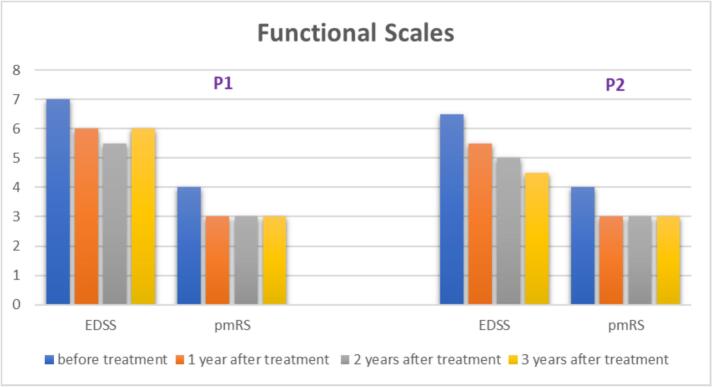
The EDSS and pmRS score, at baseline, 1 year after treatment, 2 years after treatment, and 3 years after treatment of the two patients with ASMD. In both scales, lower scores indicate less disability, whereas higher scores reflect greater disability.

Both patients maintained their functional status without deterioration after treatment. Before treatment, P1 primarily used a wheelchair and had an EDSS score of 7 and a pmRS score of 4. After treatment, P1 remained dependent on caregivers for daily activities such as toileting but was able to walk short distances with substantial assistance. The EDSS score showed a slight improvement to 6, while the pmRS score improved to 3. Before treatment, P2 did not use a wheelchair but could only walk a few meters with assistance. He had an EDSS score of 6.5 and a pmRS score of 4. After treatment, P2 was able to walk without assistance, and the distance he could walk continued to improve. At year 3 after ERT initiation, his EDSS score was 4.5 and his pmRS score was 3. The EDSS and pmRS results are shown in Fig. 3.

### 3.3 Quality of life

**Fig. 4:**
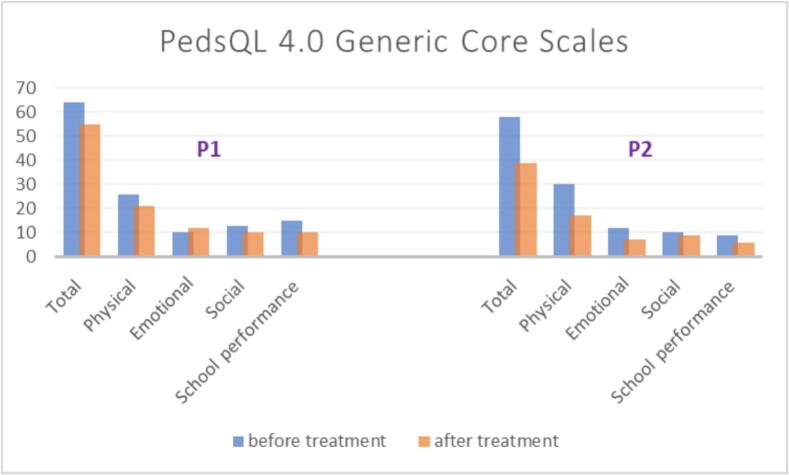
The quality-of-life score of total and each four domains provided by PedsQL™ version 4.0 questionnaire; A higher score on this questionnaire reflects a greater frequency of daily life difficulties and corresponds to a poorer quality of life.

The PedsQL 4.0 Generic Core Scales, assessing quality of life, were completed by the parents, with the results shown in Fig. 4. The total score decreased from 64 to 55 in P1 and from 58 to 39 in P2. In terms of specific domains, the physical score decreased from 26 to 21 in P1 and from 30 to 17 in P2. The social score decreased from 13 to 10 in P1 and from 10 to 9 in P2. The emotional score improved from 12 to 7 in P2 but worsened in P1, increasing from 10 to 12.

**Fig. 5:**
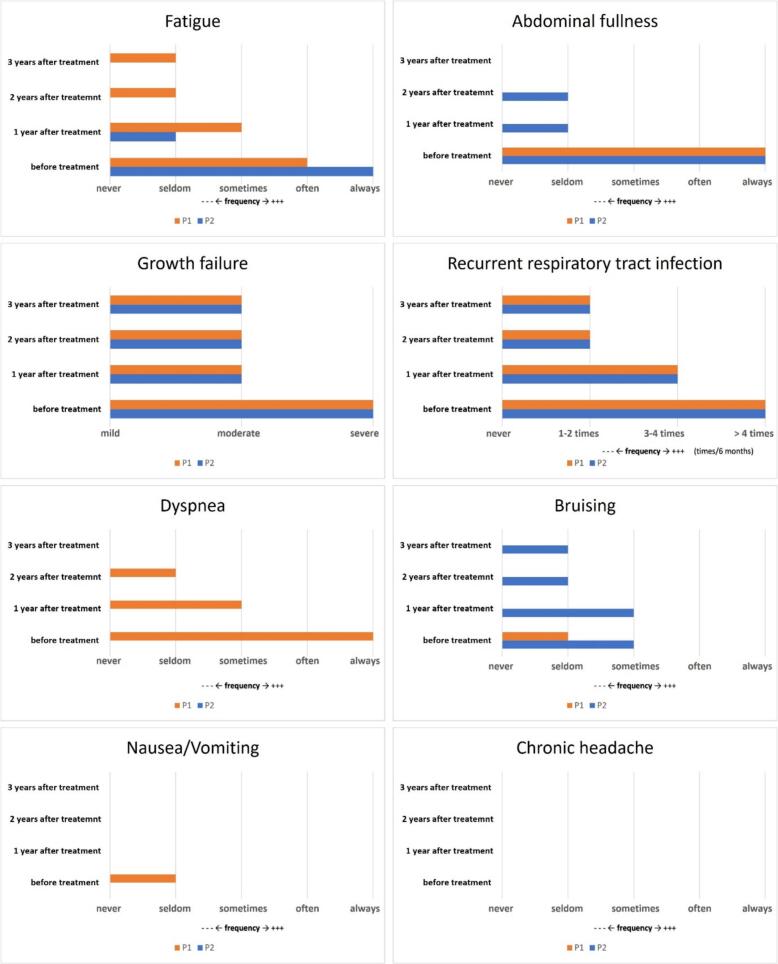
Changes in the frequency of major symptoms and signs associated with ASMD during treatment.

According to the self-designed questionnaire assessing symptom changes, Bruising, chronic headaches, and nausea/vomiting were minor symptoms in our two patients and were seldom noticed by caregivers either before or after treatment. P1 experienced significant dyspnea before treatment and showed improvement following enzyme replacement therapy (ERT). Both patients suffered from a greater frequency of abdominal fullness, fatigue, respiratory tract infection and growth failure before treatment. After treatment, the frequency decreased (Fig. 5).

## 4 Discussion

In this study, we elucidated the real-life influences of olipudase alfa on ASMD pediatric patients. First, we observed an enhancement in walking distance over six minutes for both patients following the administration of ERT. P2 exhibited a sustained improvement in walking function, whereas the walking distances of P1 appeared to stabilize after an initial increase. The different degree of improvement may be correlated with the baseline condition of the two patients. P1 and P2 share the same genotype of *SMPD1* mutations. However, there were notable differences in their baseline conditions at the start of olipudase alfa treatment. Prior to initiating ERT, P1 was wheelchair-bound, while P2 was able to walk with minimal assistance such as holding his mother's hand with one hand. Additionally, the interval between diagnosis and the initiation of ERT was longer for P1, who began ERT two and half years after diagnosis, compared to just 7 months for P2.

We further quantified functional status to evaluate the overall impact of ERT using the EDSS and pmRS. In P1, the EDSS score improved from 7 to 6, reflecting a meaningful functional gain-from wheelchair dependence prior to ERT to the ability to walk short distances with substantial assistance after treatment. P2 also demonstrated marked functional improvement, achieving independent ambulation without the need for rest during daily activities following ERT. This corresponded to an improvement in EDSS score from 6.5 to 4.5 and pmRS score from 4 to 3. Although both patients continued to require assistance with activities of daily living, including dressing, toileting, and feeding, neither required nasogastric tube feeding nor ventilatory support.

The frequency of major ASMD-related discomfort decreased over time. Caregivers of both patients reported overall improvement, with symptoms such as abdominal fullness and fatigue occurring “never” after three years of ERT. Dyspnea, which was present in P1, also resolved after treatment.

In P1, the increase in six-minute walk distance and overall functional capacity was considered to be associated with a reduction in major discomforts, including the resolution of abdominal fullness and dyspnea, as well as a decreased frequency of fatigue. Enhanced pulmonary function was also considered a contributing factor. Although formal lung function tests were not performed, serial chest computed tomography in our previous study demonstrated significant improvement in interstitial lung disease [13]. Consistent with the resolution of dyspnea, we speculate that the patient's respiratory condition improved substantially, thereby contributing to the observed gains in physical function, particularly walking ability. Similarly, although the increased walking distance in P2 may partly reflect developmental progression given the young age at treatment initiation, the caregiver reported a marked reduction in gastrointestinal symptoms and fatigue, which may also have contributed to functional improvement.

Most importantly, we observed a marked improvement in quality of life, as reflected by scores across all four domains of the PedsQL 4.0 questionnaire. Both caregivers reported that their children not only demonstrated increased physical activity but also engaged in more social interactions. Although assistance was still required for toileting and dressing, they were able to participate in simple daily tasks, such as packing their belongings or toys. With the reduced frequency and severity of discomfort and improved functional status, the patients were able to participate more actively in school and interact more with their peers. The frequency of school absence due to illness, such as respiratory tract infections, was low after three years of treatment. They were also able to participate in more school activities, which they enjoyed. In addition, they exhibited a broader range of emotional expressions, including happiness and a greater willingness to share, rather than remaining irritable due to physical discomfort. Notably, the caregiver of P1 reported an increase in emotional problems following treatment. This may be explained by P1's improved physical condition, as greater muscle strength and increased awareness of medical procedures, such as intravenous line insertion, may have led to heightened fear of needle injections.

It is important to note that ERT is not anticipated to influence neurological outcomes, as olipudase alfa, the enzyme replacement therapy, does not penetrate the blood-brain barrier (BBB). In our two patients, serial neurological examinations revealed neither signs of improvement nor rapid deterioration, supporting the clinical diagnosis of chronic neurovisceral ASMD A/B. Nonetheless, both individuals experienced significant discomfort that greatly impacted their ability to manage daily activities and quality of life.

Both patients had peripheral neuropathy at baseline, as indicated by reduced sensory and motor nerve conduction velocities. Serial nerve conduction studies performed after ERT initiation showed no significant improvement in peripheral neuropathy. Peripheral neuropathy is known to contribute to physical functional limitations [[13], [14]], as affected children may experience symptoms such as muscle weakness, numbness, pain, and impaired motor coordination, which can hinder abilities including walking, balance, and fine motor skills [16]. However, neither patient reported symptoms attributable to peripheral neuropathy, possibly because these manifestations were masked by more prominent central nervous system (CNS) involvement. Given the absence of overt clinical symptoms and the lack of improvement in nerve conduction studies, the impact of peripheral neuropathy on functional outcomes in these patients remains uncertain. Further longitudinal follow-up is required to clarify the long-term contribution of peripheral neuropathy to physical limitations and quality of life in ASMD.

Overall, although olipudase alfa does not appear to improve neurological function, including both central and peripheral involvement, it provides meaningful real-world benefits, particularly in quality of life and functional capacity, which are not directly attributable to neurological improvement.

## 5 Limitation

The study has several limitations. Firstly, the sample size is small, limiting our ability to conduct further statistical analysis. However, it is worthwhile noting that our findings align with those of the ASCEND-PED trial. Secondly, the questionnaire was filled out retrospectively by family members, introducing the possibility of recall bias. Nonetheless, previous research suggests that the impact of recall bias may not be greater than that observed in retrospective questionnaire studies [14]. Besides, the study cohort is not representative of chronic neurovisceral ASMD spectrum, given both patients had the same genotype.

## 6 Conclusion

In conclusion, although ASMD is a neurodegenerative disorder with a certain degree of neurological deterioration, and ERT is not expected to alter neurological outcomes, the general functional capability and quality of life have shown improvement in pediatric patients with chronic neurovisceral ASMD A/B after receiving olipudase alfa. Studies with larger sample sizes are needed to prove the benefits. Besides, further follow-up is necessary to monitor whether these patients experience sustained benefits in quality of life and functional status over time.

## CRediT authorship contribution statement

**Yu-Wen Pan:** Writing – original draft, Formal analysis. **Wen-Hao Yu:** Writing – review & editing, Methodology. **Meng-Che Tsai:** Writing – review & editing. **Yen-Yin Chou:** Writing – review & editing, Supervision, Resources, Methodology, Conceptualization.

## Ethics approval and consent to participate

The study was approved by the Ethics Committee of National Cheng-Kung University Hospital (approval number: A-ER-113-391). Informed consents were obtained from the legal guardian of the patients.

## Funding

This study was supported by a research grant from National Cheng-Kung University Hospital.

## Declaration of competing interest

All authors have no competing interest relevant to this article.

## Data Availability

The data that has been used is confidential.

## Appendix A Supplementary data

Supplementary material Image 1
