# Supplementary material for: Improvement in quality of life and general functions in pediatric acid sphingomyelinase deficiency patients after receiving olipudase alfa: A single-center experience in Taiwan
Source: Mol Genet Metab Rep. 2026 Apr 17;47:101312. doi: 10.1016/j.ymgmr.2026.101312 (PMC13099520; doi:10.1016/j.ymgmr.2026.101312)
Supplement: Supplementary file 1 — Supplementary material [file mmc1.docx]

Additional file 1. Results of nerve conduction velocity tests for pediatric ASMD patients

| Patient 1 | Motor nerve conduction velocity (m/s) | Peroneal nerve (L) | Peroneal nerve (R) | Tibial nerve (L) | Tibial nerve (R) |
| --- | --- | --- | --- | --- | --- |
|  | Baseline | 17 | 28 | 20 | 21 |
|  | 1 year | 22 | 20 | 23 | 22 |
|  | 2 years | 22 | 28 | 27 | 26 |
|  | 3 years | 29 | 27 | 29 | 30 |
|  | Normal cutoff values (m/s) for different age groups[1] | 2 to <3 y: 41  3 to <4 y: 43  4 to <5 y: 41  5 to <10 y: 46 | | 2 to <3 y: 42  3 to <5 y: 42  5 to <10 y: 45 | |

| Patient 1 | Sensory nerve conduction velocity (m/s) | Sural nerve (L) | Sural nerve (R) |
| --- | --- | --- | --- |
|  | Baseline | 36 | 23 |
|  | 1 year | 36 | 30 |
|  | 2 years | 44 | 38 |
|  | 3 years | 40 | 38 |
|  | Normal cutoff values (m/s) for different age groups[1] | 2 to <5 y: 50  5 to <10 y: 45 | |

| Patient 2 | Motor nerve conduction velocity (m/s) | Ulnar nerve (L) | Ulnar nerve (R) | Median nerve (L) | Median nerve (R) |
| --- | --- | --- | --- | --- | --- |
|  | Baseline | 21 | 21 | 16 | 14 |
|  | 1 year | 25 | 24 | 23 | 22 |
|  | 2 years | 37 | 38 | 36 | 33 |
|  | 3 years | 41 | 40 | 38 | 36 |
|  | Normal cutoff values (m/s) for different age groups[1] | 2 to <3 y: 45  3 to <4 y: 44  4 to <5 y: 46  5 to <10 y: 53 | | 2 to <5 y: 40  5 to <10 y: 48 | |

| Patient 2 | Sensory nerve conduction velocity (m/s) | Sural nerve (L) | Sural nerve (R) | Median nerve (L) | Median nerve (R) | Ulnar nerve (L) | Ulnar nerve (R) |
| --- | --- | --- | --- | --- | --- | --- | --- |
|  | Baseline | NA | NA | 20 | 25 | 15 | 19 |
|  | 1 year | 37 | 33 | NA | NA | NA | NA |
|  | 2 years | 39 | 40 | 33 | 38 | 32 | 31 |
|  | 3 years | 42 | 41 | 35 | 34 | 33 | 31 |
|  | Normal cutoff values (m/s) for different age groups[1] | 2 to <5 y: 50  5 to <10 y: 45 | | 2 to <3 y: 46  3 to <5 y: 52  5 to <10 y: 56 | | 2 to <5 y: 54  5 to <10 y: 56 | |

NA: Not available

[1] C.S. Ryan, E.M. Conlee, R. Sharma, E.J. Sorenson, A.J. Boon, R.S. Laughlin, Nerve conduction normal values for electrodiagnosis in pediatric patients Muscle Nerve 60 (2019) 155-160.
